# The leading role of personality in concerns about autonomous vehicles

**DOI:** 10.1371/journal.pone.0301895

**Published:** 2024-06-05

**Authors:** Márta Volosin, Martin Kálnay, Ádám Bánffi, Natália Nyeső, Gabriella Viktória Molnár, Zsolt Palatinus, Tamás Martos

**Affiliations:** 1 Institute of Psychology, University of Szeged, Szeged, Hungary; 2 Institute of Cognitive Neuroscience and Psychology, Research Centre for Natural Sciences, Budapest, Hungary; 3 Department of Ergonomics and Psychology, Budapest University of Technology and Economics, Budapest, Hungary; University of Jeddah, SAUDI ARABIA

## Abstract

Development of autonomous vehicles (AVs) is growing in a rapid rate, however, the most dominant barriers in their adoption seem to be rather psychological than technical. The present online survey study aimed to investigate which demographical and personality dimensions predict attitudes towards AVs on a Hungarian sample (N = 328). Data was collected by convenience and snowball sampling. Three-level hierarchical regression models were applied: in the first level, demographical variables, then general personality traits and third, attitude-like personality factors were entered. We demonstrated that the predictive effect of age, gender and education disappeared when personality dimensions were included into the models. Importantly, more positive general attitudes towards technology and higher optimism regarding innovations predicted eagerness to adopt AVs. On the other hand, individuals with more negative attitudes and higher dependence on technology as well as those with lower level of Sensory Sensation Seeking and higher level of Conscientiousness were more concerned about AVs. Our results suggest that AV acceptance cannot be regarded as a one-dimensional construct and that certain personality traits might be stronger predictors of AV acceptance than demographical factors.

## Introduction

The public availability of autonomous vehicles (AVs) is growing in line with the rapid rate of technological innovations. The widespread use of AVs would be promising as they have several societal and environmental benefits: besides of the increased driving safety [1–3], decreased number of traffic congestions [3–5] and reduced fuel consumption [1], AVs provide enhanced mobility for disadvantaged persons like elderly or disabled [4–7], and allow drivers higher freedom to attend to other things than driving [4]. On the other hand, the media presentations of accidents [8,9], cybersecurity issues or the lack of transparency on algorithms applied in vehicles [3,4,10], as well as the need for control during driving [10,11] are major drawbacks of acceptance of this technology.

The NHTSA (National Highway Traffic Safety Administration) classifies vehicles on a 0–5 points Likert scale based on the level of automatization. At level 0, the driver fully controls the vehicle without any automation except warning signals. Level 1 involves single autonomous assistance function (acceleration, braking, or steering) while Level 2 involves automation regarding at least two primary control functions. At level 3, the vehicle is able to drive while the driver remains available over time (conditional automation). Level 4 involves fully automated driving at a limited-service area, while at Level 5, the operation of the vehicle is not restricted (NHTSA, 2023) [12]. In spite of the spreading public availability of AV in recent days, many of Level 4 functions are still in the testing phase, and others are restricted to traffic. According to predictions, if AVs are be proven to be effective, the first highway lines could be opened in the 2050s, the roadway design and practices may start to change in the 2060s, and AVs are going to be appropriate to restrict human driving only about 2070 or even later [13].

Present studies point out that societies are still neutral or even resistant towards AV [4,14–17]. The ambivalence towards innovative technological inventions might be explained by the lack of knowledge or readiness for technology in general [18–21], or by the fact that people consider the benefits and drawbacks of innovations at the same time [22]. That is, the most dominant barriers to the adaptation of autonomous vehicles seem to be more psychological than technical [23].

Besides testing AVs in the context of engineering, the present years are crucial in mapping human factors influencing attitudes towards AV technology as well, which could be incorporated into development and communication to promote acceptance [24]. Although demographical data on respondents is easily accessible and widely studied (for a recent review see: [25]), a growing number of studies aim to investigate personality traits (e.g., [26–29] as well as to contribute to attitudes towards AVs. However, as presented later in detail, the results are not equivocal. The reasons behind the diverse results are manifold. The origin country of the studies, the method of data analysis and the tools to measure a personality construct might affect the results. More specifically, general and attitude-like personality traits are usually not distinguished. While general traits can be considered as relatively stable predispositions (e.g., the Big Five dimensions), attitude-like traits such as the propensity to use technology are influenced by learning and other situational contexts. This hierarchical combination of the broad and specific personality dimensions has been not only found to be a reliable approach in consumer research in general [30] but successfully predicted trust in AVs [29]. Therefore, we also apply this theoretical framework of personality in our study by distinguishing general personality traits and domain-related attitudes as predictors of AV acceptance.

Furthermore, even though most studies involve populations from economically developed countries, cultural differences [14] and the economic status of the given country still might lead to variability in readiness and attitudes [15,24,31]. Data on Central or Eastern-European countries are also scarce [16,21,32].

Therefore, the goal of the present study was to identify personality-level predictors (i.e., general traits and attitudes) of acceptance of AVs in a Hungarian sample. We will also take into account demographical characteristics such as gender, age, and level of education, that have previously been shown to be associated with AV acceptance. Controlling for these variables can test the predictive power of personality factors. In the following section, we review previous research by enumerating demographical and personality factors that have been found to affect attitudes towards AVs or might be potential predictors of AV acceptance.

### Demographical factors

#### Gender

One of the most typically studied demographical factors is gender, and results typically point out that men have more positive attitudes towards AVs than women in general [2,4,10,17,33,34]. Men were also more likely to use AVs [35,36] and forward collision warning [37], and more willing to relinquish control over driving [18]. On the other hand, women were found to be more concerned [18], and they rated traffic situations including AVs much riskier than males [2].

Gender differences might be linked to the fact that men tend to take higher risks at roads [2,37] and that they have more salient attitudes towards technology in general [38]. Hohenberger et al. [39] emphasized that gender differences originate from affective reactions: they found that women felt less pleasure towards AVs and felt more anxious about those while men exhibited an entirely opposite tendency; however, this difference decreased with age. Besides, women were less willing to allow their children to ride in a driverless school bus than men which was also mediated by emotional factors [40]. Finally, and as a more general explanation, cars, trucks and other motor vehicles are traditionally linked to masculinity, leading to higher pleasure towards automated systems in men [41]. Only the minority of studies failed to demonstrate the effect of gender (e.g., [20]), which might be due to the small sample size consisting of university students [26] or due cultural differences in the studied populations [14].

#### Age

The relationship between age and AV acceptance is less straightforward. Comparing cohorts from different generations, Lee et al. [42] demonstrated that age negatively impacted perception and interest in AV technology, and the youngest generation (millennials) showed the highest intentions to use it when becomes available. A linear trend was found between age and attitudes towards AVs indicating that hostility increases with age [15], which is in line with results on less positive attitudes of older respondents [2,31]. Besides, older adults were more concerned about AV technology [18] and older age predicted enhanced need for control during driving [26].

Although older persons were found to be more hesitant, according to some studies they were not more hostile than younger ones [10,43]. The age-related gap mentioned above might decrease as younger adults start to express more concerns [44], especially in the developed countries [10], and consider the benefits and obstacles of AVs at the same time [22]. Studies that found no effect of age (e.g., [20,21,24,35]) also support this notion.

In addition, there is data suggesting that older adults have greater intentions toward using AVs [18,45] and automated airport shuttles [46]. Older adults demonstrated higher acceptance of driving assistant systems as well [37], probably as they experienced more difficulties during driving.

### Residential conditions

Beside of age and gender, residential conditions also might influence attitudes towards AVs and innovations. Numerous studies suggest that individuals residing in urban areas show more positive attitudes towards AVs [15,24,33] and higher willingness to pay for them [47]. According to the study of Nielsen & Haustein [34], the most enthusiastic respondents were the younger, highly educated men living in large urban areas.

These findings are in line with other studies tracking the spread of different technologies. For example, modeling the path of Twitter usage, Toole and colleagues [48] demonstrated that the platform was started to be used by residents of college towns and metropolitan areas, followed by individuals from suburban and rural areas. Similar results were found in Hungary: the very first adopters of a popular social media platform (iWiW) were settled in towns with large population; residents of smaller towns, especially those more distant from the capital (Budapest) were rather late adopters of the platform [49].

### Education

In general, education level was found to play a significant role in AV acceptance. Higher educated individuals were more likely to adopt AV technology and were willing to pay more for it [14,15,17,33,34,47]. As Nordhoff et al. [24] emphasize, residents from countries with higher income have better English skills which might influence results from a questionnaire asked in English in a non-English-speaking country. Higher education levels in general might also be related to better English skills in these countries which are essential when interacting with a device with menus and interface in English, strengthening the accepting attitudes of higher educated people towards AVs and technology in general. Other studies failed to demonstrate the effect of education level on AV acceptance [20,36,50]. It is important to note that the majority of studies were not representative regarding education (for example university students or individuals recruited online), age or gender [21] which might lead to biased results.

### General personality factors

#### The ‘Big Five’ traits

The Five Factor Model is one of the most widely used models on the dimensionality of personality [51]. It suggests that personality can be described by five basic traits (commonly referred to as the ‘Big Five’); Extraversion, Neuroticism, Agreeableness, and Conscientiousness, and Openness. Individuals characterized by a high level of Neuroticism are perceived as anxious and prone to worry and to handle stress poorly, while a high level of Extraversion is associated with high level of energy and enjoyment of being in crowded social situations. High Openness is characterized by being curious and open to new experiences, and people with low Agreeableness are skeptical and suspicious, putting their interests before others’. Finally, individuals with higher level of Conscientiousness are achievement-oriented, cautious, dutiful, orderly, self-disciplined, and prone to self-efficacy [51]. Importantly, these traits were found to be associated with acceptance of AV technology; however, the results are not entirely equivocal.

Several studies point out that Neuroticism is an essential factor when investigating the role of personality in AV acceptance. It was demonstrated that a high level of Neuroticism (i.e., a lower level of emotional stability) negatively impacted the perceived usefulness and acceptance of AV technology [18,28,52]. Drivers characterized by a higher level of Neuroticism worried more about the security of their personal data while those with a higher level of Agreeableness were less concerned about data transmission [10]. Openness was associated with higher willingness of information sharing regarding AVs [26], higher willingness to relinquish driving control [18], and more knowledge resulting in higher acceptance [28]; however, opposite effects of Openness were also demonstrated [27]. Extraversion and Conscientiousness were found to be positively correlated with the need for control, resulting in lower acceptance of AVs [18,26,27]. Agreeableness appeared to positively affect technology acceptance attitudes, as highly agreeable individuals are more prone to being influenced by the media and their social environment [52], but at the same time it was also associated with enhanced worry about the ease of use and automation reliability [28].

#### Sensory Sensation Seeking (SSS)

Sensory sensation seeking can be defined as a personality trait which manifests in enhanced need for novel, varied experiences, and individuals with a high level of Sensory Sensation Seeking are more willing to take risk for the sake of such experiences [53]. Higher level of Sensory Sensation seeking was found to be positively correlated with risk-taking in driving, and with higher intention to use driver-assistance [54] and AV technology [18,43]. On the other hand, it was also suggested that individuals who are characterized by a higher level of Sensory Sensation Seeking are less motivated to use AVs, as they expect a more novel experience by controlling the car than being a passenger in an AV [19].

#### Locus of control

Another personality trait that might be predictive of acceptance of AV is Locus of Control, which is associated with people’s attribution of the causes of events happening around them. Individuals characterized by an internal Locus of Control tend to attribute the outcomes to their actions and abilities. On the other hand, individuals with external Locus of Control believe that outcomes are rather the results of factors which are out of their control and caused by circumstances, luck or fate [55].

Choi and Ji [19] found that individuals with external Locus of Control were more eager to use AV as they believed that safe drive rather depends on external conditions than on the driver’s abilities which led to higher intention to use AV. This also implicates that external Locus of Control is associated with enhanced trust in AV systems, especially when somebody experiences difficulties with driving [19]. Locus of Control was associated with driving behavior as well: individuals who reported higher level of internal locus of control in driving situations were more likely to exceed speed limit especially at higher speed levels [56]. On the other hand, it was also found that Locus of Control did not influence attitude towards autonomous technology [43].

#### Ego-resilience

Finally, it is important to mention Ego-resilience as a potential personality characteristic which might influence acceptance of AVs but which is less studied in context of this topic. Ego-resilience is a capacity that enables individuals to adapt to constantly changing environmental demands [57]. As technological innovations change and grow quickly, it appears reasonable to expect that individuals who can flexibly adapt to their environment and to new situations are more open to accepting AV technology. When participants were passengers in an AV driven by a human driver or in autonomous mode, Palatinus et al. [58] found that the eye movement pattern of individuals with higher level of Ego-resilience was not affected as greatly by the difference between human and autonomous driving conditions compared to those with lower level of Ego-resilience. This suggests that a higher level of Ego-resilience was accompanied by lower alertness or arousal when the vehicle was driving without human control, which might be explained by better and more flexible adaptation to the unfamiliar driving situation.

### Attitude-like personality factors

#### Readiness for technology and general attitudes towards technology

Individuals can be characterized by a general attitude towards using technology and innovations, which might significantly contribute to their acceptance of AVs. The general attitude toward technology might be assessed, for example, by Technology Readiness Index (TRI [59]) or Technology Adaptation Propensity (TAP [60]) scales, which measure distinctive dimensions of approaching and withdrawing attitudes toward technology. As these specific attitudes are relatively stable attitude-like dimensions of the personality, they be regarded as separate personality characteristics (e.g., [61,62]).

Utilizing TRI and TAP scales, it was demonstrated that individuals who were more optimistic about technological innovations were keener to use online banking systems [63] or smartphones [64]. Similarly, managers and owners of small and medium enterprises who used e-commerce were found to be more open to innovations [65]. Furthermore, individuals who were characterized by a higher level of discomfort and insecurity about technology were less motivated to use smartphone applications [66].

Regarding autonomous vehicles, individuals with higher scores on TRI showed a higher willingness to use AVs [67]. Studies utilizing other questionnaires than TRI or TAP pointed out that technology-savvy respondents who used a smartphone and who were familiar with car-sharing had a more positive attitude toward automated car sharing [47] and towards AVs in general [50], which is in line with the notion that these tech-savvy individuals are likely to be the early adapters of AV technology [33].

### AV acceptance in Hungary

Of the above-mentioned research lines, Hungarian studies usually focus on two larger areas influencing AV acceptance: demographical [68–71] and attitude-like variables [21,72–75]. These studies demonstrated that women are less optimistic regarding AVs than men [68–71,73], and younger age [68–70], higher level of education [68,70], settlement with larger population [68], and higher income [68] are also associated with higher acceptance level of AVs.

On the other hand, studies on attitudinal factors revealed that the strongest predictors of intention to use of AVs were positive attitudes towards technology, social influence and safety [72], and that hedonistic motivation increased while technology anxiety reduced intention to use of AVs [21]. Similarly, Kovács & Lukovics [75] demonstrated the high impact of expected advantages, enthusiasm on technology on AV acceptance. In addition, trust in performance and privacy [74], external driver locus of control and desire for control [32] as well as openness to tourism usage of AVs [76] were also found to be important factors in AV acceptance and intention to use. In a sample of university students (N = 1273), benefits in usefulness and situations, commonality and system concerns, as well as the optimism factor of TAP influenced AV acceptance, and importantly, these attitudinal factors fully explained gender differences as well [73].

### Aims and hypotheses

Personal attitudes toward specific technological advancements, such as AVs, are considered the results of a complex, multilevel system in personality (e.g., [29]). Therefore, the goal of the present study was to investigate how demographical, general and attitude-like personality factors together predict attitudes towards fully automated AVs. Beside the most commonly studied factors such as age, gender, education as well as Sensory Sensation Seeking or Big Five traits, we included Ego-resilience and general attitudes towards technology in our models as they were found to be correlated with different phenomena which might be associated with the acceptance of AVs.

Based on the literature presented above, we developed the following hypotheses: being male [H1; cf., 2,4,10,17,33–36,39,41], having higher level of education [H2; cf., 14,15,17,33,34,47], living in higher population area [H3; cf., 15,24,33,34,47–49], and having general personality traits such as higher level of Openness [H4; cf., 18,26,28], Agreeableness [H5; cf., 51], Sensory Sensation Seeking [H6; cf., 18,43,54], Ego-resilience [H7; cf., 58] and attitude-like personality traits such as general readiness for technology [H8; cf., 33,47,50,63–67,73] will positively contribute to the attitudes towards AVs. On the other hand, we also expected that older age [H9; cf., 2,15,18,31,42], higher level of Extraversion [H10; cf., 18,26,27], Neuroticism [H11; cf., 10,18,28,52], Conscientiousness [H12; cf., 18,26,27] and lower level of external Locus of Control [H13; cf., 19,32,56] will negatively affect attitudes towards AVs.

As literature appears to be more consistent regarding the effect of demographical factors than of personality-related individual differences, we conducted hierarchical linear regression analyses. The first model contained demographical variables (gender, age, education, population of residence), then in the second step, we added general personality traits (Extraversion, Neuroticism, Agreeableness, Conscientiousness, Openness, Ego-resilience, Locus of Control, Sensory Sensation Seeking), and finally, attitude-like personality traits (TRI and TAP scales) were included. We chose to apply 3-level hierarchical modeling because of three reasons. First, most previous studies used demographical predictors together with general personality or attitude-like trait predictors, therefore the effects of these three types of factors are intermixed. Second, the distinction of the general and attitude-like personality factors might also provide a more accurate picture on individual differences in AV acceptance. Third, general personality dimensions were not studied yet in Hungarian sample.

## Materials and methods

### Participants and procedure

328 adult volunteers participated in the study (199 women, 129 men). The mean age was 36.98 years (median = 33; SD = 14.317; 19–71 years). Most of the respondents were residents of Hungary (n = 318; 97%) and some of them were Hungarian minorities of Serbia (n = 8; 2.4%) or Romania (n = 2; 0.6%). Regarding education, only one person finished elementary school only (0.3%), 12 persons (3.7%) finished vocational training, 146 persons (44.5%) graduated from high school. 97 persons (29.6%) achieved bachelor’s degree, 58 (17.7%) persons achieved master’s degree and 14 persons (4.3%) had post-gradual degree. The population data of each settlement was based on the latest census of population on the 1^st^ of January 2019 in Hungary (Hungarian Central Statistical Office), and the census of population of 2011 in Serbia (Statistical Office of the Republic of Serbia) and Romania (National Institute of Statistics). The distribution of total population of settlements was the following: below 10000 (n = 72; 22%), 10001–100000 (n = 45; 13.7%), 100001–1000000 (n = 149; 45.4%), more than 1 million (n = 62; 18.9%). The mean of population was 4100311, and the median was 160766 (ranging from 687–1752286). Please note that the population of Budapest, the capital of Hungary is about 1750000 and the population of the second largest city of Hungary is about 210000 residents. 266 respondents owned a valid driving license (81.1%), and 313 respondents had never travelled in an autonomous vehicle before (95.4%).

Respondents were recruited online by convenience and snowball sampling via e-mails and social media and they got no monetary compensation for participation, therefore it cannot be regarded as representative and is not generalizable for the Hungarian population. The questionnaire started after they gave informed consent by accepting terms and conditions preceded by a detailed description of the goals of the study and of their rights. Filling out the questionnaire took about 20–25 minutes. Data was collected anonymously, and no participant could be identified. They could withdraw participation at any time during filling the questionnaire, and in this case, their responses were not recorded. The study was conducted in accordance with the Declaration of Helsinki and the protocol was approved by the United Ethical Review Committee for Research in Psychology, Hungary (EPKEB; Nr. 2020–89).

#### Measures

The questionnaire started with questions regarding demography (e.g., gender, age, settlement, education level etc.), followed by items on attitude towards AVs and technology, and finally, personality questionnaires were presented. The utilized scales are listed below.

#### Attitudes towards autonomous vehicles

To assess participants’ attitudes towards AVs, we translated items of the questionnaire utilized by Charness et al. [18] to Hungarian. The scale consists of 16 items, starting with an inquiring whether respondents have ever heard about the self-driving technology before. Two further questions are asked on a 10-point Likert-scale (e.g.,” What is the likelihood that you would ride as a passenger in an autonomous or self-driving vehicle, assuming that it would be at no additional cost over other driving options including driving a personally owned vehicle?” where 1 is “highly unlikely” and 10 is “highly likely”) while the rest of the items are asked in the form of a 5-point Likert scale (e.g.,” I would be comfortable with driving or riding in an autonomous or self-driving vehicle.” where 1 is “strongly agree” and 5 is “strongly disagree”). Charness et al. [18] declared three factors after the exclusion of the first item: Concern with AV (e.g., “I would be concerned with self-driving vehicles moving by themselves from one location to another while unoccupied”), Eagerness to adopt AV (e.g., “I would be comfortable with driving or riding in an autonomous or self-driving vehicle.”) and Relinquish driving control (e.g., “Computers are capable of the same quality of decision making as human drivers”).

#### Technology Readiness Index (TRI)

Technology Readiness Index measures [59] the eagerness to adapt and use technological innovations. It consists of 36 questions on a 7-point Likert scale which is divided into 4 factors: Optimism and Innovation sub-scales refer to drive while Discomfort and Insecurity refer to inhibitory dimensions. The factor structure of the Hungarian translation of TRI [64] suggested two factors, the first one was labeled as a positive or approach dimension (e.g., “Other people come to you for advice on new technologies”) and the other one corresponded to a negative or inhibitory dimension (e.g., “You do not consider it safe giving out a credit card number over a computer”) of technology acceptance. Higher scores indicate higher approach or increased inhibition toward technology. In the present study, we used the short Hungarian version of TRI consisting of two factors (TRI Positive and TRI negative). The Cronbach’s alpha of the TRI Positive scale was .893, and of TRI Negative scale was .838, respectively.

#### Technology Adaptation Propensity (TAP)

Similarly to TRI [59], TAP [60] also assess attitudes and willingness for adaptation of innovative technologies. The original version consists of 14 7-points Likert-scale items which were divided into 4 factors: Optimism, Proficiency, Dependence and Vulnerability, and the sum of the four sub-scales (reversing the values on Dependence and Vulnerability items) indicates the general distance from technology. In the Hungarian version [64] three factors were identified: Optimism (e.g., “Technology helps me make necessary changes in my life”), Proficiency (e.g., “I enjoy figuring out how to use new technologies”) and a concatenated Dependence-Vulnerability (e.g., “Technology controls my life more than I control technology”) factor. Higher Optimism and Proficiency and lower Dependence-Vulnerability scores indicate higher motivation to adapt technology. In the present study, the Hungarian version and the corresponding factors were utilized. Cronbach’s alpha values were .813. for Optimism, .855 for Proficiency and .732 for Dependence-Vulnerability, respectively.

#### Ego-Resiliency Questionnaire (ER89)

We used the Hungarian version [57] of the Ego-Resiliency Scale (ER89) [77] including three factors measured on a 4-point Likert scale from “Does not apply at all” to “Applies very strongly”: 1) Active engagement with the world (e.g.,”I enjoy dealing with new and unusual situations”), 2) Integrated performance under stress (e.g., “I quickly get over and recover from being startled”), and 3) Repertoire of problem-solving strategies (e.g., “I usually succeed in making a favorable impression on people”) [57]. Higher scores indicate the search for new information in everyday events, adaptive flexibility by the appropriate skills and quick recovering after stressful events, respectively. There are no reversed items, and a sum score can be calculated to measure this Ego-resilience as a general construct. Cronbach’s alpha was .768.

#### Brief 30-item Bipolar Rating Scale for the Five Factor Model of Personality (BBRS-30)

We used the Hungarian translation of Brief 30-item Bipolar Rating Scale assessing the following personality traits by a 7-point semantic differential scale: Extraversion (e.g., quiet–talkative), Neuroticism (e.g., calm–anxious), Conscientiousness (e.g., careless–thorough), Agreeableness (e.g., stubborn–flexible), and Openness (e.g., uninquisitive–curious) [78]. Each trait dimension is described as the sum of the corresponding ratings, and higher scores indicate higher levels of the relevant traits. The Cronbach’s alpha was .910 for Extraversion, .830 for Neuroticism, .817 for Conscientiousness, .696 for Agreeableness and .723 for Openness, respectively.

#### Rotter’s locus of control scale

The theory of Locus of Control assumes that people differ on attributions of the events they are experiencing [55]. We used the Hungarian adaptation consisting of 29 items [79], and the participants had to select between two statements for each question that they agreed with the most (e.g., “a. Many of the unhappy things in people’s lives are partly due to bad luck”. vs “b. People’s misfortunes result from the mistakes they make.”). In each pair of statement, either a or b statement scores 1 and another scores 0. There are six filler items measuring social desirability which are excluded from scoring. Scores of statements are summarized, and lower scores indicate internal while higher scores indicate external Locus of Control. Cronbach’s alpha was .687.

#### Brief Sensation Seeking Scale (SSS)

Zuckerman’s Sensation Seeking Scale is used widely as a self-report measure of Sensory Sensation Seeking [80], that is, individual differences in the optimal level of stimulation in terms of arousal. We used the Hungarian version of the Brief Sensation Seeking Scale that consists of 7 items. Each of the items contains two choice options and the respondents have to choose which of the options describes them better (e.g., “a. I like “wild” uninhibited parties.” vs. “b. I prefer quiet parties with good conversation”). In each pair of statement, one scores 1 and another scores 0. Scores of statements are summarized, and higher scores indicate a higher level of sensation seeking [53,80]. Cronbach’s alpha was .633.

### Statistical analysis

First, we described the factor structure of the questionnaire on attitudes towards autonomous vehicles created by Charness et al. [18] in our sample. Following Charness et al.’s [18] methods with two minor changes, we applied principal component analysis (PCA) on 15 items of the questionnaire (the first item which asked whether the respondent has ever heard about AVs was not included) with the criterion of eigenvalue > 1. As different dimensions of AV acceptance might be correlated and they are supposedly not independent from each other, we utilized Promax rotation instead of the orthogonal varimax rotation [81] as utilized by Charness et al. [18]. In order to the simpler interpretation, we reversed certain values of the scales that the higher values represent the stronger presence of the given dimension. In addition to Cronbach’s alpha, component reliability (CR) and average variance extracted (AVE) were calculated to assess internal consistency and convergent validity of AV acceptance scales [82–84].

Second, we aimed to identify significant predictors for attitudes towards AVs measured by the above-mentioned questionnaire of Charness et al. [18] by hierarchical linear regression. To examine the impact of education, the ordinal scale was converted into a dichotomous scale where 0 corresponded to education equal or lower than high school and 1 corresponded to education level equal or higher than BA degree. Because the population size of settlement was unbalanced in the sample, data was converted into a dichotomous variable were 0 corresponded to population below 100000 and 1 corresponded population above 100000. Gender was also regarded as a dichotomous variable as consisted of two categories. In the first step, demographical variables were entered into the model (Gender, Age, Education, Population of the settlement), then in the second step general personality factors (Extraversion, Neuroticism, Conscientiousness, Agreeableness, Openness, Ego-resilience, Locus of Control, Sensory Sensation Seeking), and finally, attitude-like personality factors (TRI Positive, TRI Negative, TAP Optimism, TAP Proficiency and TAP Dependence-Vulnerability) were submitted. In all steps of the model building Enter method was used. Statistical analysis was conducted in IBM SPSS Statistics (version 24.0 [85]). Finally, in order to reveal the sensitivity of our analyses on the present sample size, post-hoc sensitivity analysis was implemented using G*Power 3.1.9.4 [86]).

## Results

### Component structure of AV acceptance

Admission the 2-16^th^ items of Charness et al.’s [18] questionnaire to PCA with Promax rotation (KMO = .923; Bartlett’s Test of Sphericity: *Χ*^*2*^(105) = 3580.803, *p* < .001) resulted in three components with eigenvalues higher than 1.0. After the rotation, Component 1 explained the 53.851 of variance (eigenvalue = 8.078), Component 2 explained 8.883% (eigenvalue = 1.332) of the total variance and Component 3 explained 6.779% of the total variance (eigenvalue = 1.017). However, this component solution was re-considered because of three reasons. First, only one item (item 2) contributed to Component 3 with loading above .3; second, item 3 loaded under two components (Component 1 and Component 3) with relatively high loadings (.672 and .426, respectively); and third, the communality of item 11 loading on Component 2 was relatively low (.326).

Therefore, PCA analysis was repeated with the exclusion of items 2, 3 and 11 together in the next step. The value of KMO (.926), Bartlett’s Test of Sphericity (*Χ*^*2*^(66) = 3073.243, *p* < .001) as well as the communality of the items were satisfactory (between .489 and .859). After rotation, Component 1 explained 60.428% of the total variance (eigenvalue = 7.251) and Component 2 explained 9.763% of the total variance (eigenvalue = 1.172), resulting in a 70.2% explained variance. When considering items with factor loadings higher than absolute value of .5, items 6, 4, 7, 9, 5 (reversed), 8 (reversed) and 10 belonged to Component 1. These items were evaluated as **Eagerness to adopt AVs** (item loadings between .604 and .798; Cronbach’s alpha = .900; CR = .908; AVE = .590). Component 2 included items 15, 14, 16, 13 and 12 which indicated **Concerns about AVs** (item loadings between .757 and .858, Cronbach’s alpha = .937; CR = .942; AVE = .764). Cronbach’s alpha, CR and AVE values indicate that internal consistency and convergent validity of dependent variables can be considered as good [82–84]. Components correlated at *r* = -.685. The rotated component matrix is presented in Table 1.

**Table 1:** Rotated component matrix of the AV acceptance questionnaire on the present sample.

Item	Component 1	Component 2	Communality
6: If self-driving technology were now available as optional equipment on my next car purchase, I would buy or lease this technology.	.888		.701
7: I would buy or lease a completely autonomous vehicle (Level 4) if one were available.	.859		.688
4: I would be comfortable with driving or riding in an autonomous or self-driving vehicle.	.832		.734
9: I think advances in science and technology will allow driverless cars to be as safe as human drivers.	.754		.607
8: I think that autonomous vehicles can never be safer than those driven by humans.	-.716		.560
5: I would be concerned about driving or riding in an autonomous or self-driving technology.	-.693		.656
10: Computers are capable of the same quality of decision making as human drivers.	.590		.489
14: I would be concerned with commercial vehicles such as heavy trucks or semi-trailer trucks that are completely self-driving.		.932	.821
15: I would be concerned with public transportation such as busses that are completely self-driving		.921	.859
16: I would be concerned with taxis that are completely self-driving.		.853	.845
13: I would be concerned with self-driving vehicles moving by themselves from one location to another while unoccupied.		.836	.807
12: I would be concerned with riding in a vehicle with no driver controls available (no steering wheel, no brake pedal, and no gas pedal/accelerator).		.823	.656

Note: Extraction method: principal component analysis; Rotation method: Promax with Kaiser normalization. Component loadings below .3 are suppressed.

Importantly, as all retained items were using a 5-points Likert scale, responses on both components could be averaged. That is, participants were characterized with an average score on Eagerness to adopt AVs scale where higher values indicated enhanced eagerness and motivation to adopt AVs, while higher average scores on Concerns about AVs indicated higher concerns regarding AV technologies. Participants tended to be slightly more concerned about AVs but there was no significant difference between the level of Eagerness to adopt AVs (mean = 3.258, SD = 0.921) and Concerns about AVs (mean = 3.431, SD = 1.088): *t*(325) = 1.675, *p* = .095.

### Hierarchical linear regression models

Because PCA on attitudes towards AVs resulted in two, clearly distinguishable components, namely Eagerness to adopt AVs and Concerns about AVs, we decided to investigate the effect of predictors separately on these two components. We applied hierarchical linear regression (Enter method) because this way discrete and continuous predictors can be investigated together [87], and simultaneously examines relationships between hierarchical levels of grouped data [88,89]. For both dependent (outcome) variables, predictor variables were submitted in three levels. In the first step, demographical variables were entered into the model (Age, Gender, Education, Population of the settlement). In the following, we aimed to distinguish the effects of general and specific, attitude-like personality characteristics: in the second step general personality factors (Extraversion, Neuroticism, Conscientiousness, Agreeableness, and Openness, Ego-resilience, Locus of Control, Sensory Sensation Seeking), finally, attitude-like personality factors (TRI Positive, TRI Negative, TAP Optimism, TAP Proficiency and TAP Dependence-Vulnerability) were submitted. Dependent (outcome) variables were Eagerness to adopt AVs and Concerns about AVs, respectively.

Outliers outside 3 standard deviations were excluded from analyses as described at corresponding analyses. Descriptive statistics of the scale variables used as predictors is presented in Table 2, and Pearson correlations between dependent variables and predictors are presented in S1 Table.

**Table 2:** Descriptive statistics of the scale-level predictor variables used in the models.

	Mean	Minimum	Maximum	SD	N
Age	36.98	19	71	14.317	328
Extraversion	27.883	7	42	8.162	325
Neuroticism	21.784	6	39	6.795	328
Agreeableness	29.372	11	42	5.422	328
Conscientiousness	32.382	15	42	5.717	327
Openness	25.671	9	41	5.957	328
Ego-Resilience	40.701	23	56	5.248	328
LOC	11.853	1	21	3.758	326
SSS	2.465	0	7	1.794	327
TRI Positive	55.156	19	82	13.226	327
TRI Negative	29.509	9	56	10.211	328
TAP Optimism	19.753	4	28	4.671	328
TAP Proficiency	12.311	3	21	4.569	328
TAP Dependence	22.610	5	35	6.110	328

SSS = Sensory Sensation Seeking; LOC = Locus of Control; TRI = Technology Readiness Index; TAP = Technology Adaptation Propensity; TAP Dependence = TAP Dependence-Vulnerability.

#### Eagerness to adopt Avs

In case of Eagerness to adopt AVs, after excluding the one outlier data, the results of hierarchical linear regression indicated that in the Model 1 predictors explained 11.0% of the variance (*R*^*2*^_*adj*_ = .099; *F*(4, 316) = 9.801, *p* < .001). In Model 2 predictors explained 15.9% of the variance (*ΔR*^*2*^ = .048, *R*_*adj*_ = .126; *F*(12, 308) = 4.845, *p* < .001) while in Model 3 predictors explained 42.6% of the variance (*ΔR*^*2*^ = .267, *R*^*2*^_*adj*_ = .394; *F*(17, 303) = 13.218, *p* < .001). The value of Durbin-Watson test was good (1.990), suggesting the lack of autocorrelation in the data. However, TRI Positive scale and TAP Proficiency scales were characterized with relatively low tolerance (.233 and .274, respectively) as well as high VIF values (4.294 and 3.653, respectively) indicating multicollinearity. Therefore, as TRI Positive scale showed higher correlation with the dependent variable (Eagerness to adopt AVs), regression was conducted again with the removal of TAP Proficiency.

Now, in case of Model 1, predictors explained 11.0% of the variance (*R*^*2*^_*adj*_ = .099; *F*(4, 316) = 9.801, *p* < .001), Model 2 explained 15.9% of the variance (*ΔR*^*2*^ = .048, *R*^*2*^_*adj*_ = .126; *F*(12, 308) = 4.845, *p* < .001), and Model 3 explained 42.5% of the variance (*ΔR*^*2*^ = .266, *R*^*2*^_*adj*_ = .394; *F*(16, 304) = 14.023, *p* < .001), respectively. The value of Durbin-Watson test was 2.003. In the Model 1 containing demographical variables, Gender (β = -.240), Age (β = -.155) and Education (β = .148) were significant predictors of Eagerness to adopt AV. When including general personality traits in Model 2, Gender (β = -.193), Education (β = .135) and Ego-Resilience (β = .162) were significant predictors, and when adding attitude-like factors in Model 3, no significant effects remained from Model 1 or Model 2, but TRI Positive (β = .301) and Negative scale (β = -.288), and TAP Optimism (β = .172) were significant predictors. Thus, only H8 was supported by the data on Eagerness to adopt AVs. The detailed results of the models are presented in Table 3.

**Table 3:** Results of the hierarchical linear regression where the dependent variable is Eagerness to adopt AVs.

		B	SE(B)	β	t	p	Tolerance	VIF
Model 1								
	Constant	3.510	0.217		16.142	< .001		
	Gender	-0.448	0.102	-.240	-4.389	< .001	.942	1.062
	Age	-0.010	0.004	-.155	-2.787	.006	.910	1.099
	Education	0.272	0.100	.149	2.722	.007	.938	1.066
	Population	0.155	0.101	.082	1.534	.126	.993	1.007
Model 2								
	Constant	2.984	0.739		4.038	< .001		
	Gender	-0.360	0.107	-.193	-3.363	.001	.830	1.205
	Age	-0.007	0.004	-.109	-1.812	.071	.755	1.324
	Education	0.246	0.100	.135	2.444	.015	.900	1.111
	Population	0.121	0.101	.064	1.197	.232	.961	1.040
	Extraversion	-0.010	0.007	-.089	-1.398	.163	.679	1.472
	Neuroticism	0.002	0.009	.014	0.213	.831	.599	1.670
	Agreeableness	-0.011	0.009	-.062	-1.141	.255	.931	1.074
	Conscientiousness	-0.011	0.010	-.068	-1.133	.258	.750	1.334
	Openness	0.009	0.009	.057	0.932	.352	.728	1.375
	Ego-Resilience	0.028	0.013	.162	2.192	.029	.503	1.989
	LOC	-0.010	0.014	-.040	-0.694	.488	.835	1.197
	SSS	0.038	0.031	.075	1.239	.216	.748	1.338
Model 3								
	Constant	2.343	0.652		3.594	< .001		
	Gender	-0.135	0.096	-.072	-1.403	.162	.712	1.405
	Age	0.003	0.003	.050	0.944	.346	.684	1.462
	Education	0.031	0.088	.017	0.352	.725	.822	1.216
	Population	0.023	0.085	.012	0.271	.787	.948	1.054
	Extraversion	-0.009	0.006	-.083	-1.565	.119	.665	1.504
	Neuroticism	0.004	0.008	-.029	-0.502	.616	.584	1.711
	Agreeableness	0.003	0.008	.018	0.391	.696	.909	1.100
	Conscientiousness	-0.013	0.008	-.083	-1.633	.104	.738	1.355
	Openness	0.007	0.008	.044	0.860	.390	.725	1.379
	Ego-Resilience	0.007	0.011	.042	0.660	.510	.478	2.090
	LOC	0.009	0.012	.039	0.801	.424	.805	1.242
	SSS	0.019	0.026	.038	0.743	.458	.719	1.390
	TRI Positive	0.021	0.004	.301	4.897	< .001	.501	1.995
	TRI Negative	-0.026	0.005	-.288	-4.943	< .001	.559	1.788
	TAP Optimism	0.033	0.011	.172	3.151	.002	.637	1.569
	TAP Dependence	-0.014	0.008	-.096	-1.891	.060	.740	1.351

Note: Dependent variable was Eagerness to adopt Avs.

Population = population of the settlement; SSS = Sensory Sensation Seeking; LOC = Locus of Control; TRI = Technology Readiness Index; TAP = Technology Adaptation Propensity; TAP Dependence = TAP Dependence-Vulnerability.

Model 1 explained 11% of the variance (*R*^*2*^_*adj*_ = .099; *F*(4, 316) = 9.801, *p* < .001), Model 2 explained 15.9% of the variance (*ΔR*^*2*^ = .048, *R*^*2*^_*adj*_ = .126; *F*(12, 308) = 4.845, *p* < .001), and Model 3 explained 42.5% of the variance (*ΔR*^*2*^ = .266, *R*^*2*^_*adj*_ = .394; *F*(16, 304) = 14.023, *p* < .001), respectively.

#### Concerns about Avs

In case of AV-related concerns, after excluding the one outlier case, the results of hierarchical linear regression indicated that in the Model 1 predictors explained 8.8% (*R*^*2*^_*adj*_ = .076; *F*(4, 315) = 7.586, *p* < .001), in Model 2 predictors explained 18.7% (*ΔR*^*2*^ = .099, *R*^*2*^_*adj*_ = .155; *F*(12, 307) = 5.888, *p* < .001) and in Model 3 predictors explained 38.9% of the variance (*ΔR*^*2*^ = .202, *R*^*2*^_*adj*_ = .357; *F*(16, 303) = 12.081, *p* < .001). The value of Durbin-Watson test was good (1.997).

In Model 1 containing demographical variables, Gender (β = .216), Age (β = .133) and Education (β = -.120) were significant predictors of Concerns about AV. When introducing general personality traits in Model 2, the effects of Gender (β = .122) and Education (β = -.144) remained significant, moreover, Conscientiousness (β = .146) and Sensory Sensation Seeking (SSS) (β = -.233) appeared to be significant predictors. Finally, when attitude-like personality dimensions were also included in Model 3, the significant effects of Conscientiousness (β = .161), Sensory Sensation Seeking (SSS) (β = -.161) remained; in addition, TRI Positive (β = -.159) and Negative scale (β = .352) and TAP Dependence-Vulnerability (β = .155) also had significant predictive values on Concerns about AVs. That is, H6, H8, and H12 got supported by the data on Concerns about AVs. The results of the models are presented in Table 4.

**Table 4:** Results of the hierarchical linear regression where the dependent variable is Concerns about AVs.

		B	SE(B)	β	t	p	Tolerance	VIF
Model 1								
	Constant	3.212	0.262		12.252	< .001		
	Gender	0.477	0.123	.216	3.889	< .001	.941	1.062
	Age	0.010	0.004	.133	2.354	.019	.911	1.098
	Education	-0.260	0.120	-.120	-2.165	.031	.939	1.065
	Population	-0.193	0.122	-.086	-1.584	.114	.992	1.008
Model 2								
	Constant	2.503	0.862		2.902	.004		
	Gender	0.270	0.125	.122	2.165	.031	.830	1.205
	Age	0.003	0.005	.043	0.716	.474	.751	1.332
	Education	-0.247	0.117	-.114	-2.101	.036	.901	1.110
	Population	-0.120	0.118	-.053	-1.013	.312	.962	1.039
	Extraversion	0.016	0.008	.118	1.877	.061	.675	1.481
	Neuroticism	0.016	0.011	.098	1.470	.143	.598	1.672
	Agreeableness	0.016	0.011	.079	1.475	.141	.931	1.074
	Conscientiousness	0.028	0.011	.146	2.455	.015	.751	1.331
	Openness	-0.009	0.011	-.050	-0.826	.409	.727	1.376
	Ego-Resilience	-0.020	0.015	-.097	-1.344	.180	.505	1.982
	LOC	0.015	0.016	.053	0.933	.351	.834	1.200
	SSS	-0.135	0.036	-.223	-3.731	< .001	.743	1.347
Model 3								
	Constant	2.082	0.800		2.604	.010		
	Gender	0.104	0.117	.047	0.888	.375	.714	1.400
	Age	-0.006	0.004	-.084	-1.546	.123	.680	1.471
	Education	-0.034	0.107	-.016	-0.322	.748	.825	1.212
	Population	-0.008	0.104	-.004	-0.080	.936	.950	1.053
	Extraversion	0.012	0.007	.087	1.578	.116	.659	1.518
	Neuroticism	0.008	0.009	.049	0.838	.402	.583	1.715
	Agreeableness	0.004	0.010	-.020	0.429	.668	.910	1.099
	Conscientiousness	0.031	0.010	.161	3.085	.002	.741	1.350
	Openness	-0.006	0.010	-.031	-0.589	.557	.724	1.381
	Ego-Resilience	-0.009	0.013	-.042	-0.646	.519	.482	2.077
	LOC	-0.009	0.014	-.031	-0.624	.533	.808	1.238
	SSS	-0.097	0.032	-.161	-3.023	.003	.710	1.408
	TRI Positive	-0.013	0.005	-.159	-2.510	.013	.503	1.998
	TRI Negative	-0.038	0.006	.352	5.868	< .001	.559	1.788
	TAP Optimism	-0.012	0.013	-.053	-0.949	.343	.642	1.557
	TAP Dependence	0.028	0.009	.155	2.975	.003	.739	1.354

Note: Dependent variable was Concerns about Avs.

Population = population of the settlement; SSS = Sensory Sensation Seeking; LOC = Locus of Control; TAP Dependence = TAP Dependence-Vulnerability.

Model 1 explained 8.8% of the variance (*R*^*2*^_*adj*_ = .076; *F*(4, 315) = 7.586, *p* < .001), Model 2 explained 18.7% of the variance (*ΔR*^*2*^ = .099, *R*^*2*^_*adj*_ = .155; *F*(12, 307) = 5.888, *p* < .001), and Model 3 explained 38.9% of the variance (*ΔR*^*2*^ = .302, *R*^*2*^_*adj*_ = .357; *F*(16, 303) = 12.081, *p* < .001).

Post-hoc sensitivity analysis indicated that the final regression models with the present sample size were able to detect effects of Cohen’s *f*^*2*^ = 0.090 (*η*^*2*^ = .083) with 95% power, suggesting that our analyses were sensitive enough to detect at least medium effects.

## Discussion

The goal of the present study was to identify demographical, general, and attitude-like personality factors predicting attitudes towards AVs in a Hungarian sample. Although the level of Eagerness to adopt AVs and Concerns about AVs were similar in the sample, hierarchical linear regression models revealed that slightly distinctive factors predicted these two dimensions of AV acceptance. Individuals who showed higher approach and lower inhibition towards technology measured by TRI and those who were more optimistic about technology were more willing to use AVs. On the other hand, concerns were also predicted by TRI scales and respondents with higher level of dependence on technology and Conscientiousness, as well as those with lower level of Sensory Sensation Seeking were more concerned about AVs.

When investigating the dimensionality of AV acceptance, two components were identified as a result of PCA, suggesting that it can be regarded as a construct characterized with more than a single dimension. Indeed, this is in line with most studies which defined several aspects of acceptance and attitudes towards autonomous technology from 2 to as high as 10 (e.g., [10,18,19,26,90]. The variability in the number of measured dimensions across the literature might arise from different theoretical and methodological practices, further indicating that there is no uniform way to assess the complexity of AV acceptance.

Regarding personality dimensions, general attitudes towards technology measured by TRI Positive and Negative scales predicted both AV-related eagerness and concerns. In addition, higher level of TAP Optimism predicted higher level of eagerness, and higher level of TAP Dependence-Vulnerability predicted more concerns, supporting H8. This suggests that individuals who are more tech-savvy and more optimistic about technological innovations are more likely to accept AVs, and that anxiety about negative impacts of technology like overdependence or being controlled by it strongly contributes to the enhanced AV-related concerns. The fundamental role of optimism in the perception of future technologies [91], expected advantages and enthusiasm for new technologies [16] were also demonstrated on Hungarian samples. These findings resonate to previous results on positive relationship between attitudes towards technological innovations in general and AV acceptance [29,33,50,67].

In addition to the technology-related personality traits, concerns about AVs were predicted by Sensory Sensation Seeking and Conscientiousness as well, as predicted by H6 and H8. Respondents who reported lower level of Sensory Sensation Seeking and higher level of Conscientiousness were more concerned about AVs. These results are in correspondence with previous findings showing that individuals who were less open to novel experiences or to risk-taking behaviors were less motivated to use AVs [18,43], and that a conscientious person worries more about giving up control over the vehicle [18,26,27]. Interestingly, while in case of Eagerness to adopt AVs the predictive value attitude-like personality traits dominated, individual differences in general personality dimensions were more pronounced in concerns. This partial dissociation further supports the above-mentioned notion on the multi-dimensionality of AV acceptance and suggests that at least partly different factors contribute to the levels of Eagerness to adopt and Concerns about autonomous technology.

On the other hand, Extraversion (H10), Neuroticism (H11), Agreeableness (H5), Openness (H4), Locus of Control (H13) and Ego-resilience (H7) failed to appear as consistent significant predictors, therefore these hypotheses were rejected by our data. In the study of Kyriakidis et al. [10], Neuroticism was associated with worry about data security issues in particular and not to a more general dimension of AV acceptance or avoidance as measured in the present study. We found no significant effect of Extraversion neither on Eagerness or Concerns about AVs, which is in line with the results of Amichai-Hamburger et al. [26]. The null effect of Agreeableness can be explained by findings that point out that although agreeable individuals’ attitudes were more likely to be influenced positively by their environment [51], they were also more worried about the reliability of automation [28]. Contrary to our hypothesis and to previous findings [18,26,27,92], Openness also failed to be a significant predictor which is in line with Kyriakidis et al.’s [10] results. The lack of a significant effect of Locus of Control corresponds to Payre et al. [43], who suggested that external Locus of Control during driving might be associated with the need to use technology in order to compare the individual’s and system’s skills, leading to null effect. Finally, although Ego-resilience was expected to be positively associated with attitudes towards AVs, it was not found to be significant in any of the final models.

Personality traits, such as the five-factor model’s dimensions and the other broad personality factors in our study, are supposed to represent a person’s generalized response tendencies to a broad range of real-world situations [93]. However, our results provided evidence that these general factors explain little variance in how people relate to specific but little experienced phenomena, such as AVs, especially compared to established technology attitudes. This may mean that more contextualized and domain-specific experiences need to be considered when explaining attitudes toward AVs [29]. Nevertheless, the relative inability of general traits to predict these attitudes may inform researchers and practitioners that these attitudes are not “hard-wired” in the basic structure of the personality and, thus, can be subject to change.

We hypothesized that, in addition to personality dimensions, demographical variables are going to predict attitudes towards AVs as well. Contrary to our predictions, gender (H1), age (H9) and education (H2) showed significant effects in the Model 1s only, that is, when no personality dimensions were included. That men and younger respondents were more eager to adopt and less concerned about AVs fits the literature (e.g., [2,18,33]. In addition, corresponding to former results, individuals who completed at least secondary school were more eager to use AVs [14,15,17,33,34,47], which might be due to the fact that the result of higher education level being more likely to correlate with advanced English skills, which are necessary to interact with different devices [24]. However, when further variables were included into the model, these significant effects disappeared, thus, H1, H2, H3 and H9 were not supported by the data anymore.

In further steps we investigated the predictive effects of general (Extraversion, Neuroticism, Conscientiousness, Agreeableness, and Openness, Ego-resilience, Locus of Control, Sensory Sensation Seeking) and attitude-like (TRI Positive, TRI Negative, TAP Optimism, TAP Proficiency and TAP Dependence-Vulnerability) personality dimensions separately in addition to demographical variables. When including general personality traits into model, only Ego-resilience appeared to be a significant predictor of Eagerness to use AVs, which disappeared when adding attitude-like factors, leading to the rejection of H7. In case of Concerns about AVs, on the other hand, Sensory Sensation Seeking and Conscientiousness were significant predictors, and this effect did not change with the presence of attitude-like personality dimensions in the model, in other words, H6 and H8 and H12 were supported while H4, H5, H7, H10, H11, and H13 were rejected by the data. This is in line with results of Kraus et al. [29], who demonstrated that both elemental and dispositional levels of personality traits predicted trust in AVs. This also suggests that although attitude-like personality dimensions might correlate at some level with general traits, they are more accurate predictors of AV-related concerns and eagerness.

Our results regarding the weak explanatory power of demography after including personality dimensions into the regression models resonate to the literature. Similar patterns of results were demonstrated in studies utilizing linear regression methods. For example, when both personality and demographical factors were included into the model predicting the willingness to share information, stepwise method excluded age and gender while the effect of Openness remained significant [26]. More importantly, in correspondence with the present study, in a hierarchical regression model the significant predictive value of age and gender disappeared when general and attitude-like personality factors were entered in further steps [43]. Also note that p values depend on the sample size [94], and in the present study, post-hoc sensitivity analysis revealed that the final models were able to detect medium or large effects only, therefore it is possible that a larger sample size would have distinguished small effects as well.

When comparing results from different studies, besides the obvious methodological differences, cultural characteristics [14] and the economic status of a given country should be taken into consideration, as these contextual factors also contribute to variability in attitudes towards AVs [15,16,21,24]. The different rate of the development of AV technology across the world also results in different perceptions in distinctive regions of the world as well as diversities of societies’ readiness [10]. The results of the present study reflect the attitudes of a Hungarian sample where level 2 of automation is legally permitted to use in traffic. According to the latest KPMG report of 30 countries, Hungary was ranked to the 25^th^ place on the Autonomous Vehicle Readiness Index while for example Singapore, USA, or South Korea where numerous studies were conducted were ranked to the 1^st^, 4^th^ and 7^th^ place, respectively. Furthermore, the digital skills of consumers, the level of government’s readiness for change, the efficiency of the legal system in challenging regulations, as well as the transparency of data-sharing environments were ranked among lowest in Hungary [31]. This is in line with the results of Majó-Petri and Huszár [72], who revealed that although 97% of their Hungarian respondents have already heard about AVs, only 23% of them look up information on this technology regularly.

The present study has certain limitations. First, although one advantage of the online studies is that they allow a higher diversity of the sample with respect to demographical variables [39], convenience and snowball sampling might lead to bias as individuals who are more familiar with online technology as well as those who are interested in the topic of automated driving are more prone to fill out the survey. Second, and accordingly, gender, education and population size of the settlement were not perfectly balanced within this non-representative sample, its results cannot be generalizable for the whole population. Third, in the present study, all variables were assessed by psychological scales using self-reported data, and reliance on self-reports might be a source of bias in understanding the phenomena under study [95]. Fourth, as the present study was conducted on a Hungarian sample only, no direct comparison was possible with international data. A fifth and a more general limitation of the survey studies regarding AV acceptance is that most of the respondents had no prior experience with autonomous driving systems; therefore, their responses can be regarded as mostly hypothetical. It was demonstrated that being a passenger in an autonomous vehicle changed attitudes toward AVs [22,90,96], and increased the explanatory power of psychological models of acceptance [97,98].

Future studies need to reach a wider, more heterogeneous sample of respondents from countries with different economic and cultural backgrounds to make direct comparisons and put the results in a more accurate and wider context. Regarding methodological issues, results could be improved in numerous ways. First, based on the results of the present study, not only the direct effects of predictors, but hypothesis-driven mediation or moderation analysis could be used. Second, combining survey data with allowing participants to experience AVs in a controlled experimental design could also contribute to better reliability of results of self-reported questionnaires [98]. Third, qualitative methods such as interviews or narrative texts could provide essential information on reasons for mapping consumers’ concerns and motivations regarding AV from further different perspectives as well. Fourth, beside demography and personality, identifying social [99], cognitive or emotional factors [100] would also shed more light on the nature of AV acceptance. Finally, it is also important to note that the results may have been influenced by newly translated scales like the BBRS-30, which currently have limited validity data. These considerations call for replication with other measures.

In addition to its contribution to the current line of research on attitudes towards AVs, the present study has several implications for applied science. In contrast to the relatively easily identifiable demographical characteristics, mapping attitudinal and personality dimensions require more effort and complex methods. Nevertheless, by combining these different types of information and differentiating general and attitude-like personality traits, a more sophisticated profile might be assessed of respondents, which allows approaching consumers in a more personalized way and increasing their engagement, for example, in marketing and information campaigns [29]. Such campaigns might be also helpful before experiencing AVs, for example by admitting ambivalent aspects of the technology or developing users’ competences. Similarly, as the spreading of innovations can be regarded as progress based on communication [70] and is largely impacted by social influences [99], considering individual differences can be also beneficial for building communicational strategies on AVs, both in industrial and governmental [73] as well as in touristic [76] aspects.

In summary, the aim of the present study was to identify demographical and personality factors that predict attitudes towards AVs. Regarding personality dimensions, general approaching and withdrawing attitudes towards technological innovations predicted both eagerness to use AVs and concerns about AVs. In addition, individuals more optimistic about technological innovations were more willing to use AVs, and those characterized by a lower level of Sensory Sensation Seeking, a higher level of Conscientiousness and a higher Dependence on technology were found to be more concerned about AVs. That concerns and motivations regarding to the use of AVs were associated with at least partly distinctive factors strengthens the assumption that acceptance of AVs cannot be regarded as a one-dimensional construct. Finally, as certain personality traits were found to be stronger predictors of attitudes towards AVs than demography per se, it might be crucial to take individual differences into consideration in AV acceptance research.

To conclude, as the development of AVs intensifies, mapping attitudes towards self-navigating technologies becomes a challenge. The groundwork for identifying factors that may signal approach or distancing, such as our study, is inevitable at the current stage. Our study aimed to contribute to this area of research by including demography and personality factors at once, and in addition by differentiating general and attitude-like personality traits, allowing us to get a more sophisticated picture on AV acceptance. Consequently, a more elaborate and perhaps more precise strategy will require a coordinated effort between developers and human behavior researchers that examines how specific features of the AVs affect people’s perception and attitudes.

## Supporting information

S1 Table Pearson correlation coefficients between dependent (outcome) variables and predictor variables. Population = population of the residential area; SSS = Sensory Sensation Seeking; LOC = Locus of Control; TAP Dependence = TAP Dependence-Vulnerability. p < .05*; p < .001**.
(DOCX)
