# Supplementary material for: The leading role of personality in concerns about autonomous vehicles
Source: PLoS One. 2024 Jun 5;19(6):e0301895. doi: 10.1371/journal.pone.0301895 (PMC11152294; doi:10.1371/journal.pone.0301895)
Supplement: S1 Table — Population = population of the residential area; SSS = Sensory Sensation Seeking; LOC = Locus of Control; TAP Dependence = TAP Dependence-Vulnerability. p < .05*; p < .001**. (DOCX) [file pone.0301895.s001.docx]

**Supplementary Information**

**S1 Table.** Pearson correlation coefficients between dependent (outcome) variables and predictor variables.

|  | Eagerness to adopt AVs | Concerns about AVs | 1 | 2 | 3 | 4 | 5 | 6 | 7 | 8 | 9 | 10 | 11 | 12 | 13 |
| --- | --- | --- | --- | --- | --- | --- | --- | --- | --- | --- | --- | --- | --- | --- | --- |
| Eagerness to adopt AVs |  |  |  |  |  |  |  |  |  |  |  |  |  |  |  |
| Concerns about AVs | -.725** |  |  |  |  |  |  |  |  |  |  |  |  |  |  |
| 1 Age | -.181** | .163** |  |  |  |  |  |  |  |  |  |  |  |  |  |
| 2 Extraversion | -.028 | .026 | .139* |  |  |  |  |  |  |  |  |  |  |  |  |
| 3 Neuroticism | -.006 | .053 | -.190** | -.451** |  |  |  |  |  |  |  |  |  |  |  |
| 4 Agreeableness | .097 | .111* | -.015 | .017 | -.114** |  |  |  |  |  |  |  |  |  |  |
| 5 Conscientiousness | -.109* | .179** | -.192** | .268** | -.377** | .118* |  |  |  |  |  |  |  |  |  |
| 6 Openness | .165* | -.150** | -.143** | -.145** | -.035 | -.028 | -.056 |  |  |  |  |  |  |  |  |
| 7 Ego-Resilience | .159** | -.126* | .053 | .454** | -.487** | .078 | .298** | .421** |  |  |  |  |  |  |  |
| 8 LOC | -.151** | .148* | -.024 | -.133* | .219** | .130* | -.058 | -.184** | -.315** |  |  |  |  |  |  |
| 9 SSS | .201** | -.311** | -.362** | .154** | .007 | -.076 | -.161** | .197** | .172** | -.069 |  |  |  |  |  |
| 10 TRI Positive | .517** | -.331** | -.202** | .141* | -.148* | -.099 | .035 | .175** | .316** | -.190** | .167** |  |  |  |  |
| 11 TRI Negative | -.490** | .535** | .264** | .026 | .062 | .151** | .054 | -.139* | -.126* | .245** | -.292** | -.341** |  |  |  |
| 12 TAP Optimism | .412** | -.229** | -.172* | .084 | .012 | -.106 | .018 | .129* | .165** | -.127* | .096 | .551** | -.274** |  |  |
| 13 TAP Proficiency | .403** | -.247** | -.351** | .064 | -.055 | -.098 | -.019 | .195** | .225** | -.098 | .234** | .818** | -.269** | .432** |  |
| 14 TAP Dependence | -.195** | .275** | -.025 | -.088 | .178** | .018 | -.132* | -.077 | -.128* | .172** | -.066 | -.028 | .403** | .032 | .059 |

Population = population of the residential area; SSS = Sensory Sensation Seeking; LOC = Locus of Control; TAP Dependence = TAP Dependence-Vulnerability

p < .05*; p < .001**
